# Bilateral acute limb ischemia complicating incessant supraventricular arrhythmias in wolff-parkinson-white syndrome: evaluating thromboprophylaxis across multiple cardioversions

**DOI:** 10.1186/s43044-025-00697-1

**Published:** 2025-10-23

**Authors:** Kellyn Trycia Zenjaya, Ragil Nur Rosyadi, Angelina Mulyadi, I Dewa Gede Nalendra Djarya Iswara

**Affiliations:** 1 Universitas Hang Tuah, Surabaya, Indonesia; 2 Dr. Ramelan Naval Center Hospital, Surabaya, Indonesia; 3 Dr. Ramelan Naval Center Hospital, Surabaya, Indonesia

**Keywords:** Acute limb ischemia, Arrhythmia, Thromboprophylaxis, Wolff Parkinson white syndrome, Case report

## Abstract

**Background:**

Acute limb ischemia (ALI) is a severe complication in patients with structural heart disease and recurrent arrhythmias that often leads to significant morbidity and potential lifelong disability. The combination of arrhythmia, recurrent electrical shocks in patients with incessant supraventricular tachycardia (SVT), and structural heart abnormalities may increase the risk of thrombus formation and migration, raising questions about the role of thromboprophylaxis in such high-risk scenarios.

**Case presentation:**

We present a 54-year-old female with Wolff-Parkinson-White (WPW) Syndrome and structural heart disease who was admitted with incessant SVT, complicated by several episodes of ventricular tachycardia (VT) and ventricular fibrillation (VF). Despite multiple unsynchronized cardioversions, the patient remained hemodynamically unstable. Echocardiography revealed a dilated left ventricle with reduced systolic function and an intracardiac thrombus. During hospitalization, she underwent over ten direct current (DC) shocks, exacerbating her thromboembolic risk. On day three of hospitalization, she developed severe pain in her right leg, progressing to signs of ALI. Doppler ultrasound and CT angiography confirmed thrombi in the abdominal aorta and lower extremities, supporting the diagnose of ALI Rutherford III dextra and ALI Rutherford IIA sinistra. Surgical thrombectomy and anticoagulation were initiated, but ALI progressed, necessitating above-knee amputation in her right leg.

**Discussion:**

Thromboprophylaxis in patients with structural heart disease and incessant arrhythmia must be carefully assessed. Incessant SVT alone does not justify early anticoagulation, but coexisting cardiomyopathy and repeated cardioversions increase thromboembolic risk. Identifying the underlying cause of structural abnormalities is essential to guide treatment and prevent severe complications. Delayed anticoagulation in the presence of undetected thrombus may result in irreversible events such as limb loss due to ALI.

## Background

Acute limb Ischemia (ALI) is a critical vascular condition that results from a sudden reduction in blood flow to the limb leading to limb amputation or death. It commonly arises from in situ thrombosis, especially in patients with peripheral arteries disease or those who have had vascular procedures such as stenting or bypass grafting, followed by the progression of atherosclerotic arteries. Cardioembolic events due to conditions such as arrhythmia, recent myocardial infarction and valvular heart disease also account for many cases [1, 2]. Patients with arrhythmias, including those with Wolff-Parkinson-White (WPW) syndrome, are at particular risk.

WPW syndrome is a congenital arrhythmia involving an accessory pathway that bypasses the AV node, leading to AVRT in 55% of cases [4]. This pathway can cause wide QRS complexes on ECG and conduct atrial arrhythmias such as fibrillation or flutter, resulting in rapid ventricular rates. Ventricular arrhythmia, therefore, can increase the thromboembolic risk especially in patients with myocardial infaction [3]. This case presents bilateral acute limb ischemia as a complication in a patient with incessant SVT, structural heart disease, and a history of multiple cardioversions.

Interventions like cardioversion and antiarrhythmic medications, although necessary, can increase the risk of thromboembolic events, further complicating the management of ALI [4]. Thrombolysis can be a therapeutic option with acceptable results in patients with ALI but may cause significant clinical complication such as systemic or intracranial bleeding [5]. The timing of cardioversion and the use of thromboembolic prophylaxis should be carefully considered, as they may be contraindicated based on the patient's condition.

This case highlights the importance of thromboprophylaxis in high-risk patients with recurrent arrhythmias and structural heart disease. Despite aggressive management, including anticoagulation and surgical intervention, the patient’s condition progresses. This calls attention to the need for individual approaches to anticoagulation therapy in reducing thromboembolic risk.

## Case presentation

A 54-year-old woman presented to our hospital with worsening palpitations, chest pain, and shortness of breath over the previous two weeks. Initial evaluation on previous hospital revealed narrow-complex tachycardia at 180 bpm, indicative of orthodromic atrioventricular reentrant tachycardia (AVRT) in the context of Wolff-Parkinson-White (WPW) syndrome. The patient had received digoxin and amiodarone at the referring hospital prior to transfer. Upon arrival at our facility, despite administration of bisoprolol (BB), and amiodarone, her tachycardia persisted, and she became hemodynamically unstable with hypotension and pulmonary edema. Adenosine was not administered due to its unavailability in our country. Cardioversion at 50 to 150 J was performed, transiently restoring sinus rhythm at 74 bpm, which revealed the underlying WPW pattern on ECG.

**Fig. 1:**
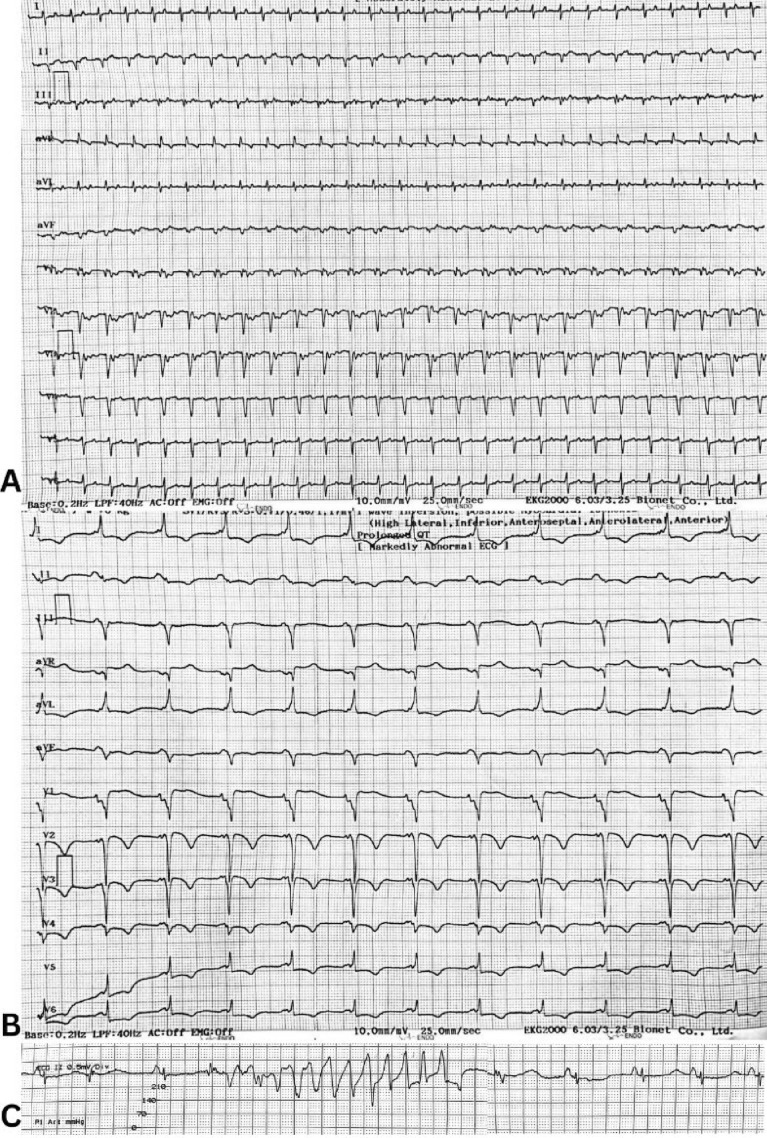
Patient's ECGs. Panel A: ECG upon arrival with narrow-complex tachycardia. Panel B: ECG after electrical cardioversion, WPW rhythm. Panel C: Torsades de Pointes

Chest X-ray showed cardiomegaly, left pleural effusion, and pulmonary edema. Blood gas analysis revealed metabolic acidosis. She was transferred to the ICU for stabilization but experienced recurrent episodes of supraventricular tachycardia (SVT) requiring additional cardioversions. Due to her refractory SVT and deteriorating clinical status, she was referred to our hospital for electrophysiology (EP) study and ablation. Upon further evaluation, she exhibited persistent tachycardia (VT) and episodes of pulseless ventricular fibrillation (VF), necessitating multiple direct current (DC) shocks. On the second day of admission, she experienced multiple episodes of VT requiring cardiopulmonary resuscitation and DC shocks at 200 J, with transient recovery of sinus rhythm. Patient’s ECG can be seen in Fig. 1. Throughout her hospitalization, continuous cardiac monitoring and serial ECGs did not show any episodes of atrial fibrillation or flutter. Definitive treatment was achieved through an electrophysiological (EP) study, which identified a right posterolateral accessory pathway as the source of the arrhythmogenic substrate. Catheter ablation of the accessory pathway was performed successfully, eliminating the underlying cause of her recurrent SVT, VT, and her hemodynamic instability. Post-procedure, the patient’s ECG showed sinus rhythm at 62 bpm, and she remained free of arrhythmias.

**Fig. 2:**
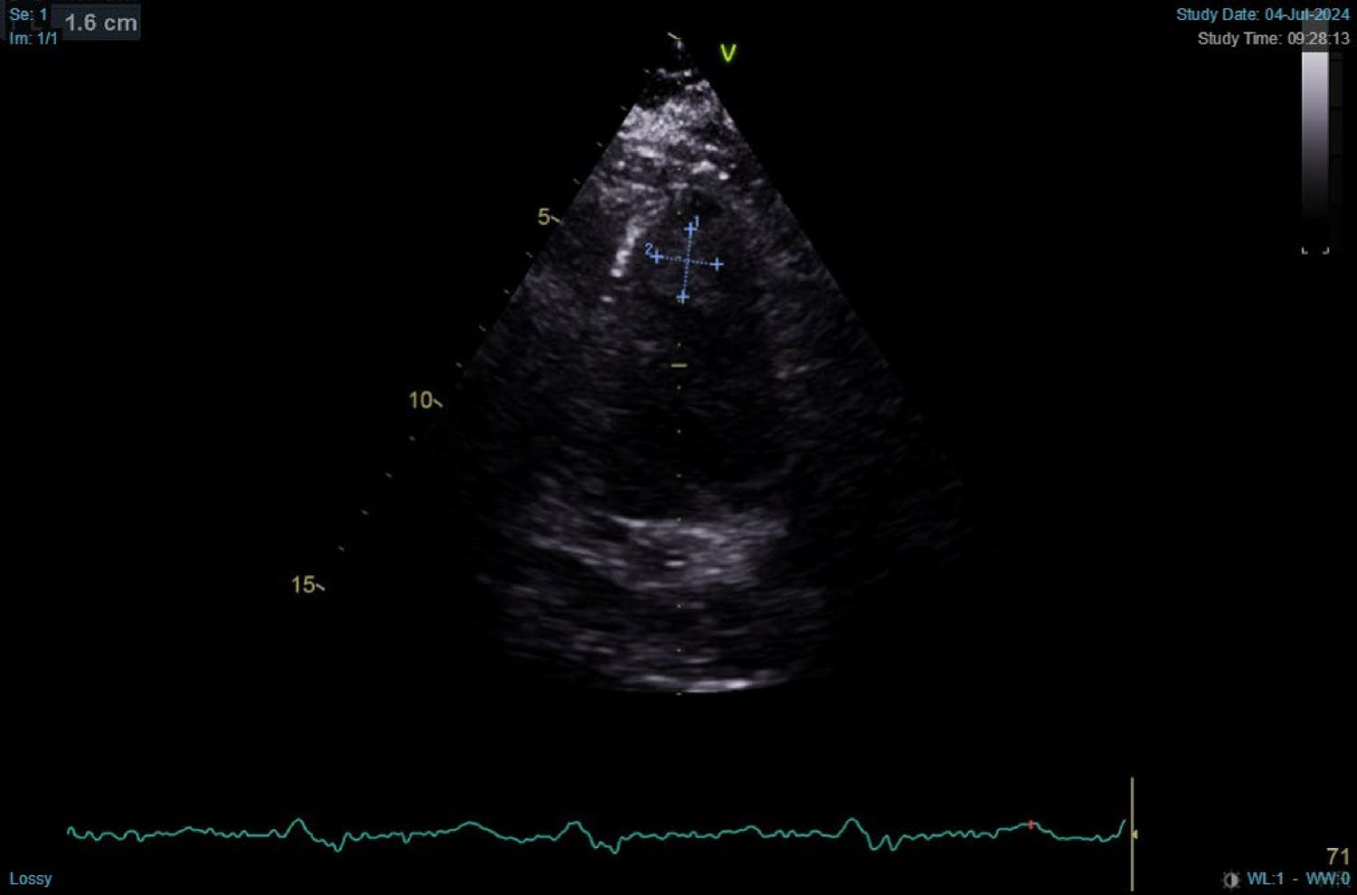
Patient’s echocardiography shows intracardiac thrombus

On the third day of hospitalization, she developed pain in her right leg. Echocardiography was performed during her 3rd days of hospitalization after her condition is stable, which revealed significant structural heart abnormalities, including mild left ventricular (LV) dilation, reduced LV systolic function (ejection fraction: 36% by Teich, 29% by biplane), concentric LV hypertrophy, and an intracardiac thrombus (1.4 × 1.6 cm) at the LV apex as shown in Fig. 2. Additional findings included akinesis of the anteroseptal wall, hypokinesis in other segments, mild pericardial effusion, and intact interatrial and interventricular septa. Transesophageal echocardiography (TEE) was not performed due to institutional limitations and lack of available resources. Anticoagulation was initiated immediately following the echocardiographic confirmation of left ventricular thrombus. The patient was started on warfarin 2 mg with bridging therapy using unfractionated heparin, which was maintained until the target INR of 2–3 was reached.

**Fig. 3:**
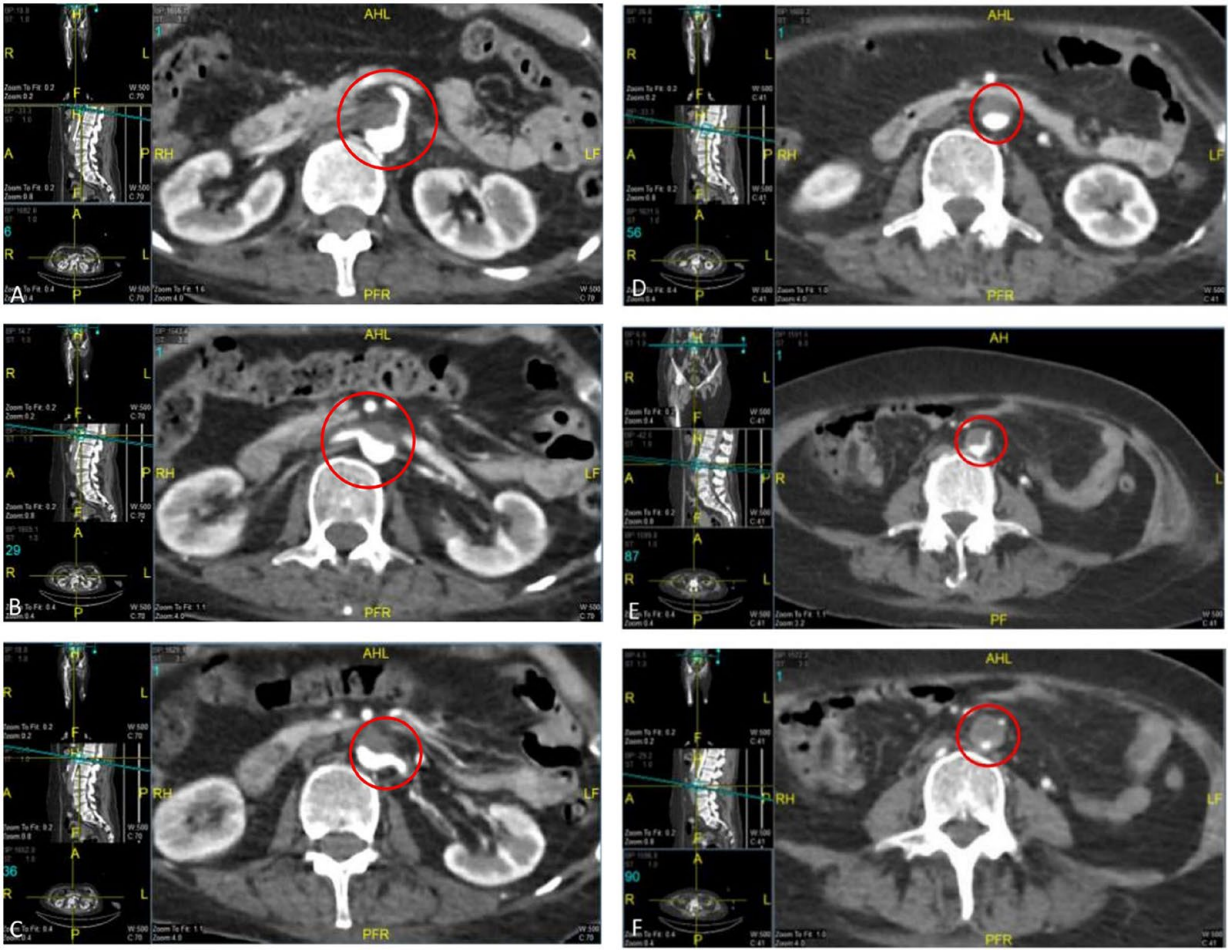
CT angiography showed thrombus at multiple sites. A, Lumbar Vertebrae level 1 distal branching with the Superior Mesenteric Artery (thrombus diameter: 1.9 cm × 1.1 cm). B, Lumbar Vertebrae level 2 cranial branching with the Right Renal Artery (thrombus diameter: 1.7 cm × 1.9 cm). C, Lumbar Vertebrae level 2 distal branching with the Left Renal Artery (thrombus diameter: 1.6 cm × 0.9 cm). D, Mid Lumbar Vertebrae level 3 (thrombus diameter: 1.6 cm × 0.6 cm). E, Lumbar Vertebrae level 4 cranial branching with the Inferior Mesenteric Artery (thrombus diameter: 1.7 cm × 0.95 cm). F, Mid Lumbar Vertebrae level 4 (thrombus diameter: 1.7 cm × 1.7 cm)

**Fig. 4:**
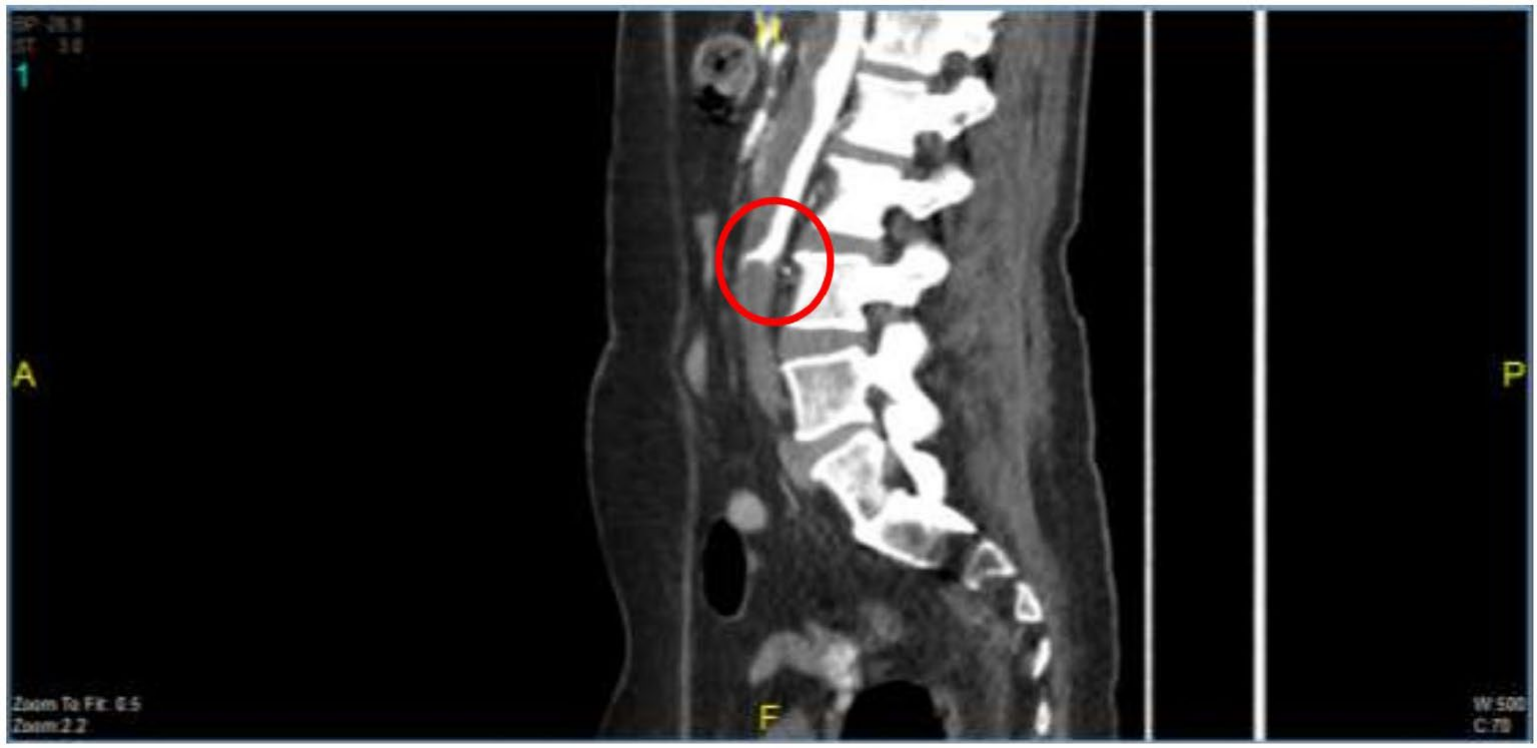
Contrast stopped at iliac bifurcation

**Fig. 5:**
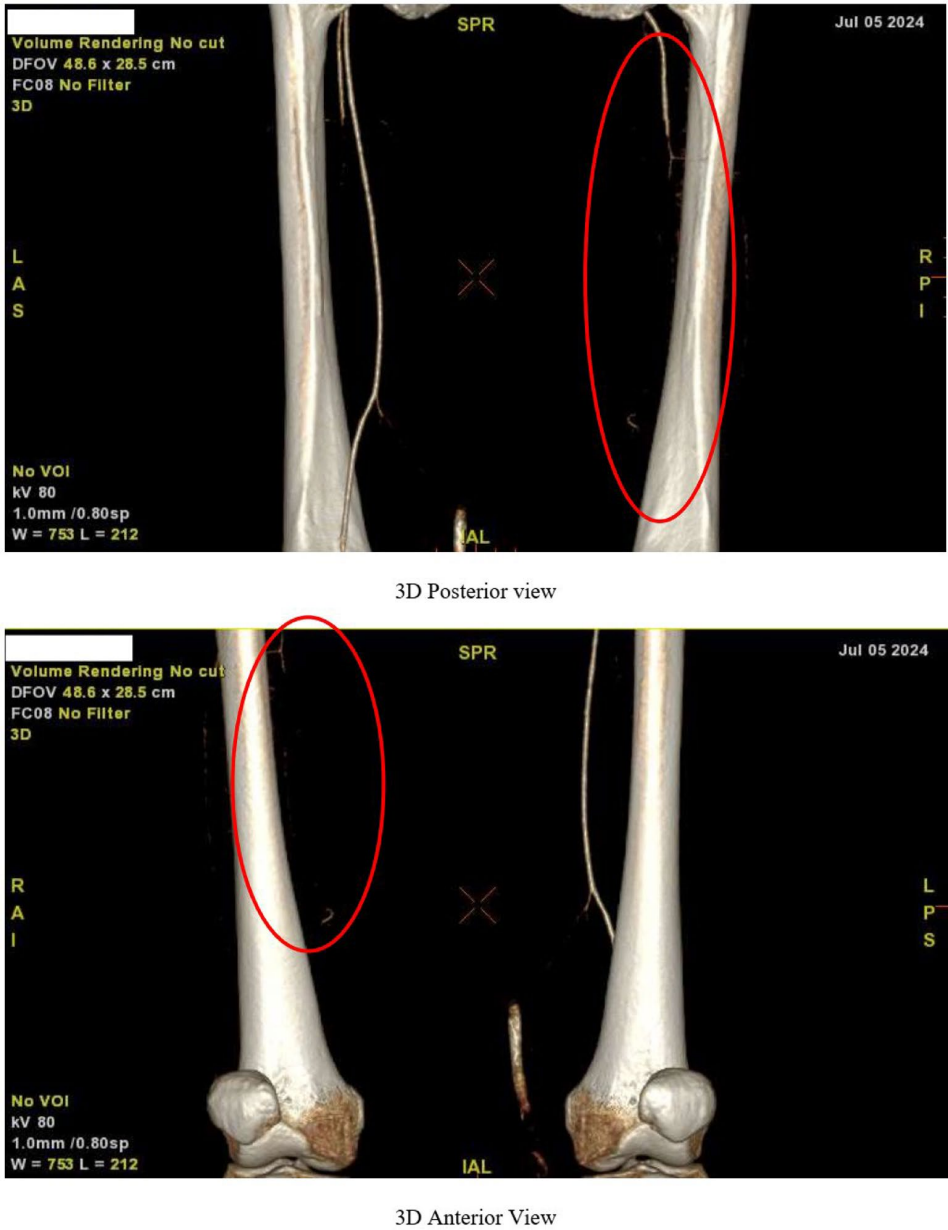
Anterior and posterior views of the right femoral artery. Contrast flow stops about 9 cm from the beginning of the right femoral artery

**Fig. 6:**
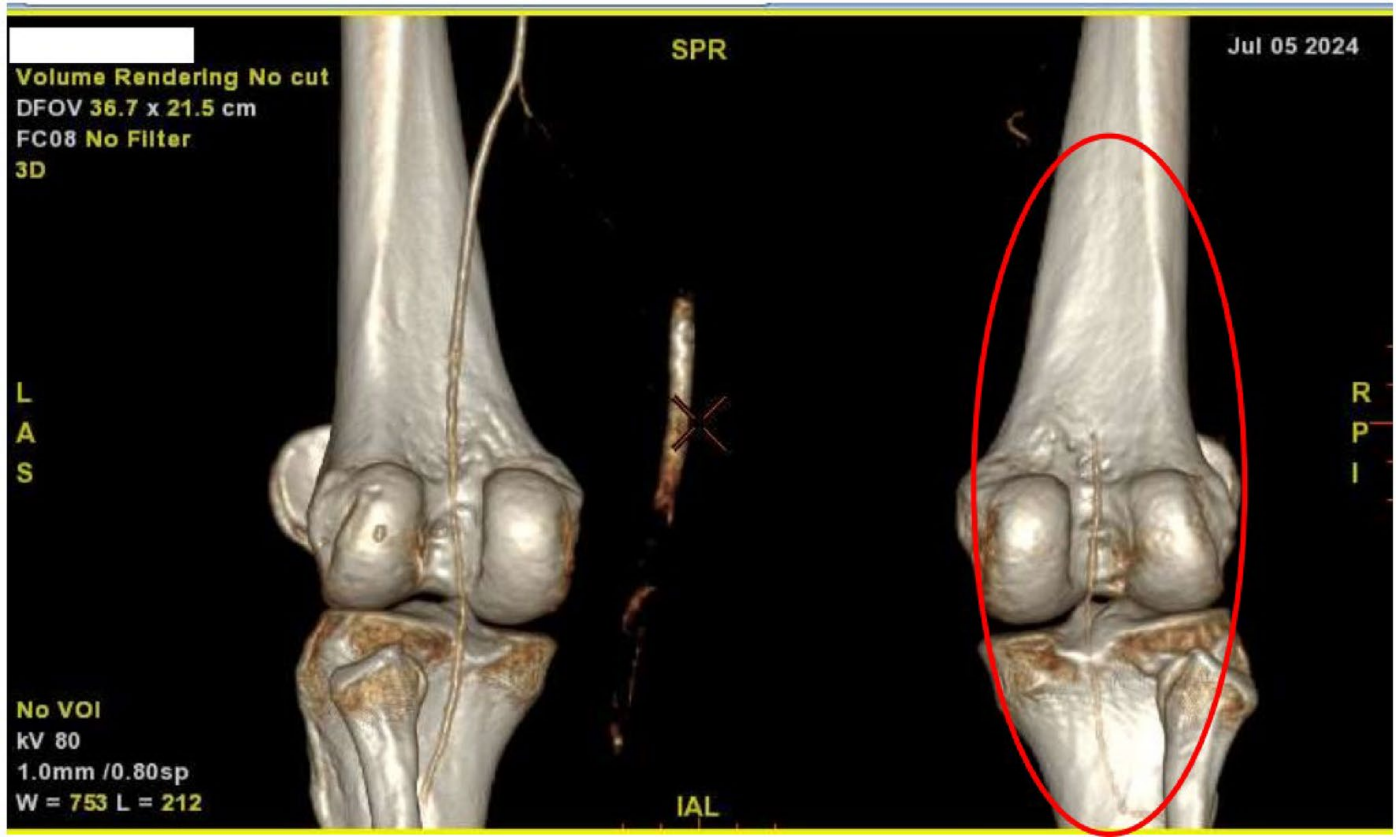
The right popliteal artery is visualized/fillied with contrast with diameter of approximately 0.22 cm

**Fig. 7:**
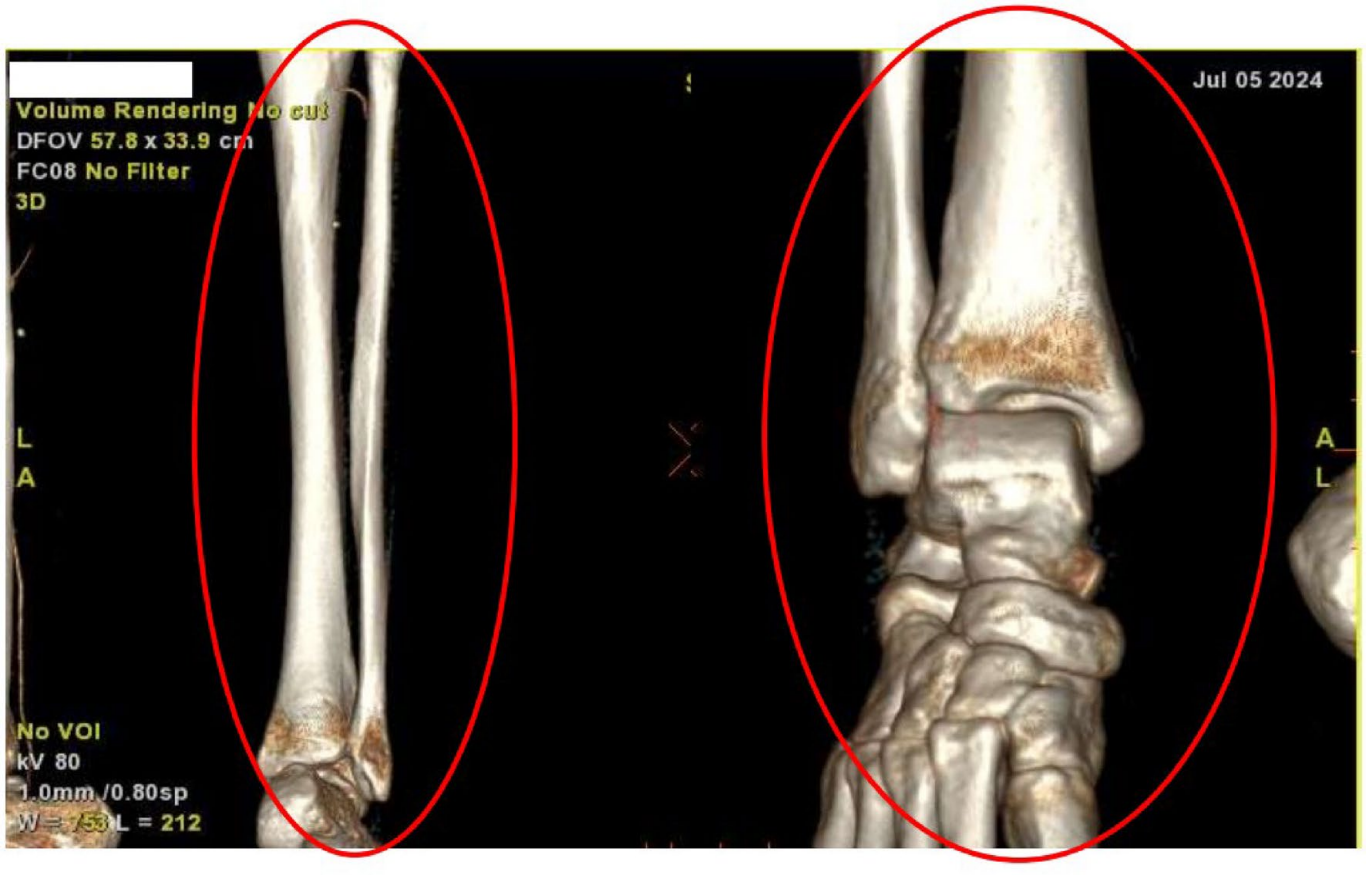
The right anterior tibial artery, the right posterior tibial artery and the right peroneal artery are not filled with contrast

**Fig. 8:**
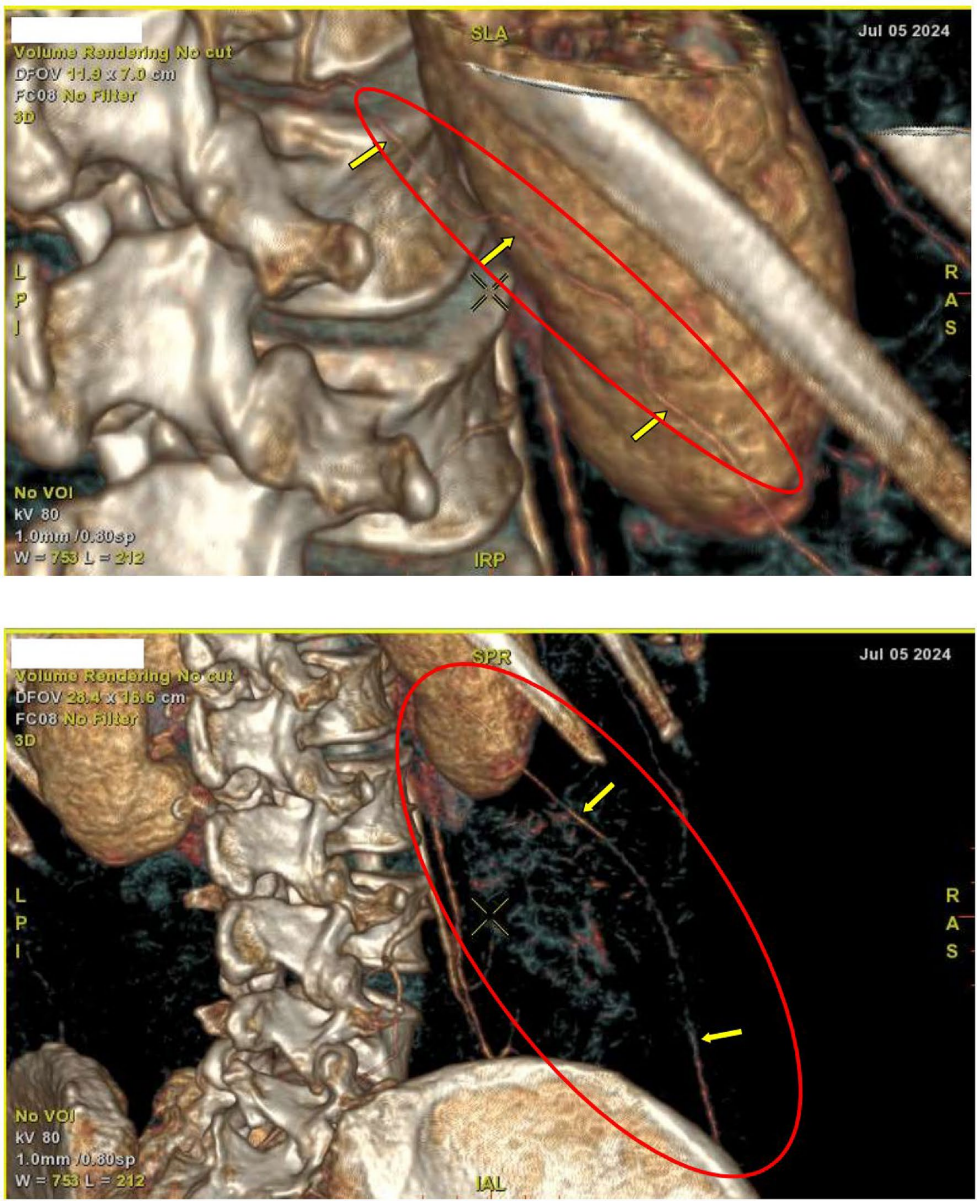
Anastomosis visualized for the right femoral region mainly from the right segmental artery at the lumbar vertebrae level 1

**Fig. 9:**
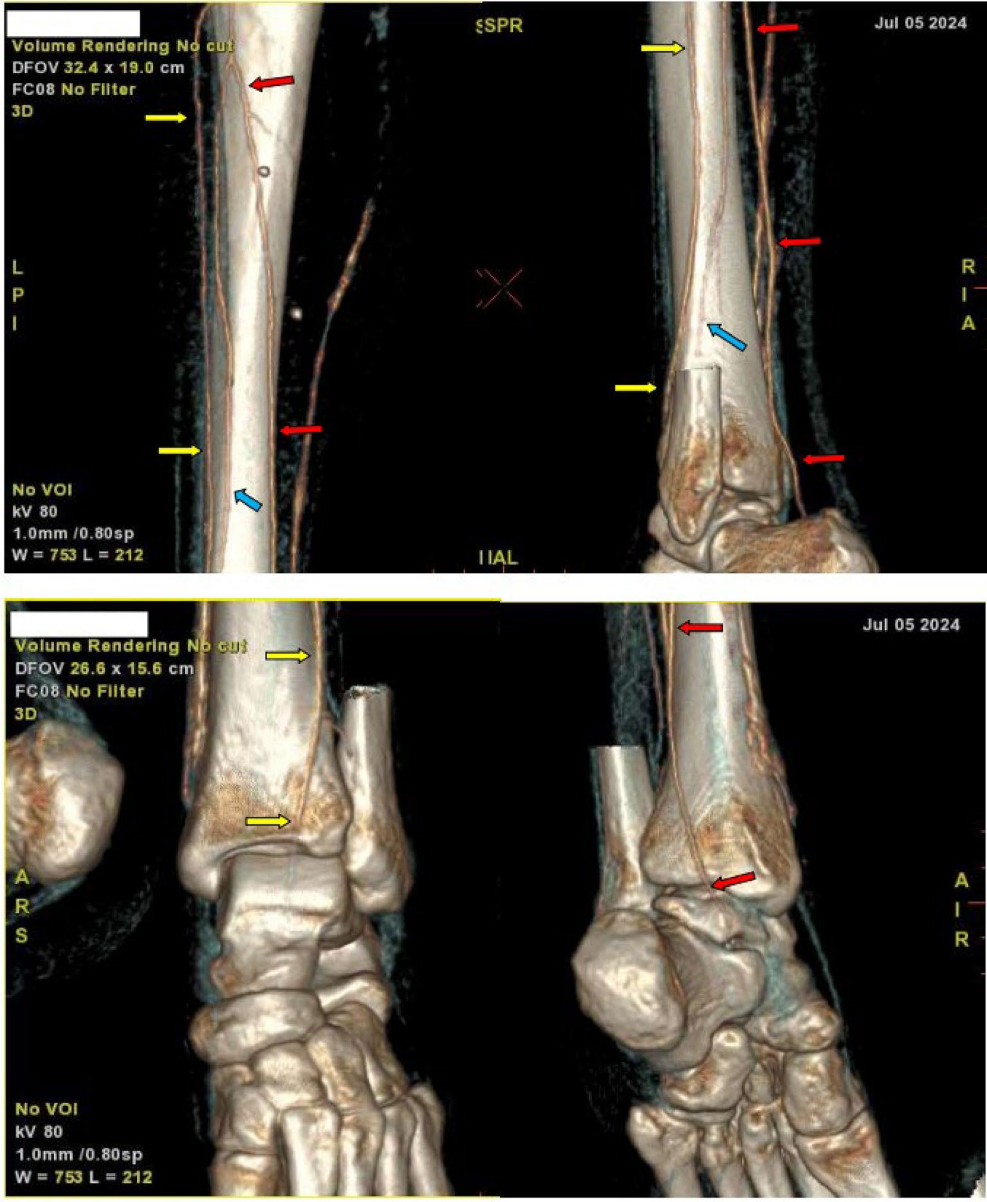
The arterial system in the left inferior extremity is well visualized up to left cruris, with thrombus causing irregularities in the true lumen surface

**Fig. 10:**
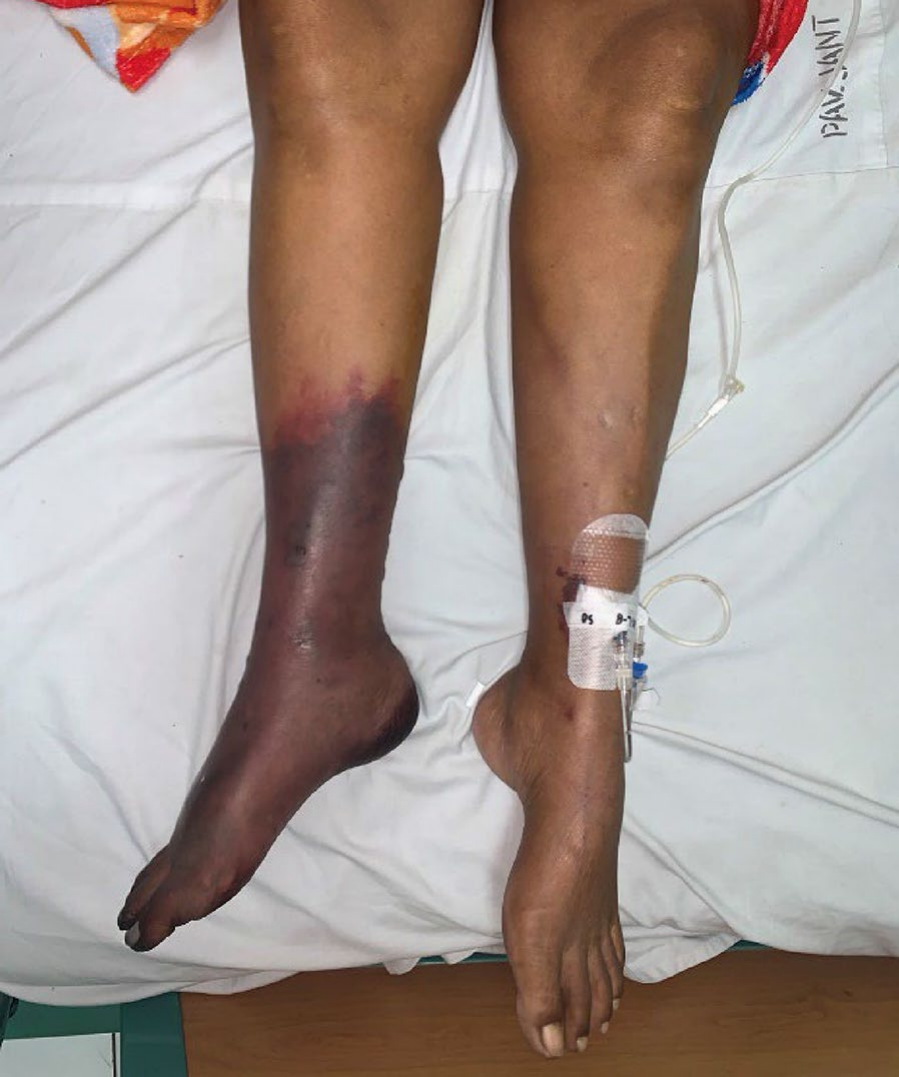
Right lower limb: ALI Rutherford III; left leg: post anterograde-retrograde thrombectomy

**Fig. 11:**
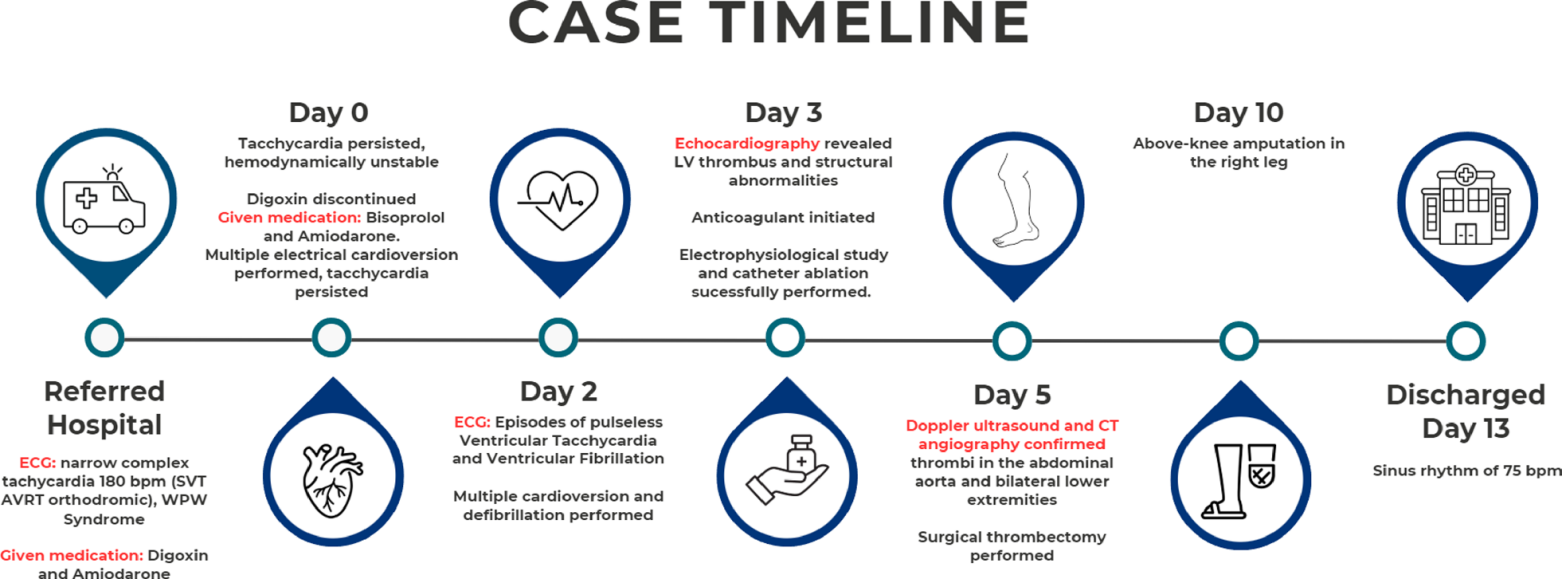
Summary timeline of clinical events, diagnostic findings, and key interventions

By the fifth day of hospitalization, her dorsum pedis showed signs of hypoxia. Doppler ultrasound and CT angiography confirmed thrombi in the abdominal aorta and bilateral lower extremities which can be seen in Figs. 3, 4, 5, 6, 7, 8, and 9. Her right lower extremity was classified as Rutherford III (nonviable limb), while the left leg was classified as Rutherford IIA (viable but threatened limb). Surgical thrombectomy was performed, restoring perfusion to the left leg. Sign of ALI in her right leg and post thrombectomy for her left leg displayed in Fig. 10. Despite heparinization and continued anticoagulation with warfarin and rivaroxaban, the right leg remained ischemic, necessitating above-knee amputation, which was carried out on the tenth day of hospitalization. A summary timeline of the patient’s clinical events, diagnostic workup, interventions, and outcomes is presented in Fig. 11.

## Conclusion

Acute limb ischemia (ALI) is a medical emergency that occurs due to the sudden reduction or cessation of blood flow to a limb, often due to embolic or thrombotic events [6]. The risk of ALI is heightened in patients with arrhythmias, especially those with underlying structural heart disease, as demonstrated in this case [7–9]. The patient, a 54-year-old woman with Wolff-Parkinson-White (WPW) syndrome, experienced recurrent episodes of SVT and worsening hemodynamic instability. The ECG (Fig. 1) shows torsades de pointes which was likely induced by prior treatment with digoxin and amiodarone, administered at referring hospital. Upon admission, electrolyte levels were within normal limits, and there was no evidence of significant hypokalemia or hypomagnesemia.

Despite the initial attempts to control the arrhythmia using beta-blockers, calcium channel blockers, and amiodarone, the patient's tachycardia persisted, resulting in critical clinical deterioration and necessitating multiple electrical cardioversions. In this patient, the structural heart abnormalities as seen in her echocardiography, contributed to the formation of an intracardiac thrombus weightened by the incessant arrhythmia. Importantly, this patient never developed atrial fibrillation during the entire hospitalization, indicating that the thromboembolic complication occurred independently.

The classical presentation in this patient includes excruciating pain, associated with pallor, pulselessness, and neurological deficit, which is sufficient to establish the diagnosis of ALI [10]. The Rutherford classification has been utilized in assessing the severity for ALI, categorizing it as viable (class I), threatened (class II), marginally threatened (IIa), immediately threatened (IIb), and irreversible damage (class III) based on vascular exam, sensory and motor deficit, and Doppler signal findings [11, 12]. Overall, ALI is often represents as a result of an underlying etiology, most commonly a cardioembolic event, which accounts for up to 60% of cases [13].

The management of incessant arrhythmias in patients with WPW syndrome is particularly challenging when complicated by thromboembolic risks, as observed in this case. The patient required multiple cardioversions to restore sinus rhythm, yet the recurrent arrhythmic episodes placed her at ongoing risk of thrombus formation and embolization. Cardioversion, though effective in restoring normal sinus rhythm, may inadvertently dislodge thrombi from the atrial walls, leading to systemic embolization [14]. Careful management of anticoagulation therapy in patients undergoing multiple cardioversions needs to be assessed, as the benefits of rhythm control must be weighed against the risks of embolism.

In this patient, anticoagulation was initiated with heparin and later transitioned to warfarin and rivaroxaban on day 3 after echocardiography confirmed thrombi in the left ventricle. Anticoagulation was not started earlier because incessant SVT, unlike atrial fibrillation, is not inherently thrombogenic and therefore does not routinely warrant prophylactic anticoagulation unless additional risk factors, such as structural heart disease or intracardiac thrombus, are identified. Despite these interventions, the patient’s right lower extremity continued to show signs of ischemia, classified as ALI Rutherford III (irreversible damage) for her right leg, and ALI Rutherford IIa (viable but threatened) for her left leg, ultimately requiring above-knee amputation for her right leg. The patient’s thromboembolic complications, including thrombosis in the abdominal aorta and bilateral lower extremities, were likely the result of a combination of factors: persistent tachycardia, the presence of an intracardiac thrombus exacerbated by multiple cardioversions, and delayed anticoagulation.

In this case, the patient’s refractory arrhythmia and the need for electrophysiology (EP) study and ablation were key to stabilizing her rhythm. However, the limited availability of advanced EP studies in Indonesia poses a significant challenge in the management of such cases. In regions with limited access to these resources, clinicians must manage these patients conservatively yet aggressively, focusing on maintaining hemodynamic stability, preventing thromboembolism, and using the tools available, such as anticoagulation and cardioversion.

This outcome reinforces the importance of timely anticoagulation and thromboprophylaxis to prevent complications such as ALI, but also highlights the limitations of current therapeutic strategies in managing high-risk arrhythmia patients. In cases like this, early initiation of anticoagulation therapy, ideally before cardioversion, is recommended to mitigate the risk of thromboembolic events. However, as this case demonstrates, achieving a balance between adequate anticoagulation and minimizing bleeding risk during frequent cardioversions remains a clinical challenge. Current guidelines for the management of arrhythmias and thromboembolic risk are valuable but may not fully address the complexities of high-risk cases such as WPW syndrome with refractory arrhythmia. As seen in this case, guidelines often provide a general framework but do not offer specific guidance for managing patients with multiple, recurrent arrhythmic episodes, requiring multiple cardioversions especially in patients with structural heart abnormalities. It is important to note that this patient had evidence of structural heart disease, as demonstrated by the echocardiographic findings. Therefore, further assessment to determine the underlying cause of the structural abnormality would have been warranted. However, in this case, a definitive etiologic evaluation could not be performed. The patient was lost to outpatient follow-up, and no repeat echocardiographic assessment was obtained to evaluate potential recovery of cardiac function.

In this case, patient came with incessant SVT and episodes of VT and VF. Therefore, the initial management strategy focused on managing the malignant arryhthmias. The discovery of the LV thrombus on transthoracic echocardiography significantly influenced the clinical course and therapeutic strategy. The apical localization of the thrombus, coupled with regional wall motion abnormalities and ECG features suggestive of anterior wall infarction, raises strong suspicion for an underlying ischemic event, likely an anterior myocardial infarction. Unfortunately, coronary angiography could not be performed due to limitations in healthcare funding and national insurance coverage (BPJS). Nevertheless, the presence of LV thrombus necessitated immediate anticoagulation, in accordance with current guidelines. In retrospect, given her ischemic ECG pattern, cardioversions, earlier empiric anticoagulation might have been justified for anticipating thromboembolic risk even before imaging confirmation.

Based on guidelines by American College of Cardiology/American Heart Association (ACC/AHA) and European Society of Cardiology (ESC) guidelines about managing supraventricular and ventricular arrhythmia, there is no specific recommendation of using anticoagulation in preventing thromboembolic events as these arrhythmias are not independently associated with thromboembolic risk [15]. The guidelines primarily focused on atrial fibrillation (AF) due to its high prevalence and well-documented association with thromboembolic risks, particularly stroke and systemic embolism [16–20]. In our case, the patient had pre-existing structural heart disease, evidenced by impaired left ventricular systolic function on echocardiography prior to the onset of incessant tachyarrhythmia, and was also known to have WPW syndrome. The sustained supraventricular arrhythmia may have exacerbated the cardiomyopathic state, but the thromboembolic event was likely precipitated by the underlying cardiomyopathy rather than by the arrhythmia itself. Anticoagulation in such cases is guided not by the arrhythmia itself but by the presence of structural abnormalities and thrombus. According to the 2022 AHA Scientific Statement on the management of left ventricular thrombus, therapeutic anticoagulation is strongly indicated in patients with confirmed LV thrombus, regardless of the underlying etiology [21]. In our case, warfarin with heparin bridging was initiated upon echocardiographic diagnosis of thrombus. For other arrhythmias requiring cardioversion, such as persistent VT or SVT refractory to medical therapy, anticoagulation is typically not a routine recommendation unless thrombus is identified via imaging or pre-existing risk factors. However, the decision to initiate anticoagulation is based on multiple factors, including the age, duration of the arrhythmia, presence of structural heart disease, and underlying comorbidities [16, 18, 20]. Anticoagulation agents like warfarin, direct oral anticoagulants (DOACs) (e.g., apixaban, rivaroxaban, dabigatran), or aspirin (in lower-risk scenarios) are used, with the choice depending on the patient's risk profile and other clinical considerations. Scoring system such as CHA₂DS₂-VASc Score (primarily to assess stroke risk in patients with AF) and HAS-BLED score have been widely used to guide the decision to start anticoagulant therapy. HAS-BLED score are more commonly used for assessing bleeding risk associated with anticoagulation therapy, while CHA₂DS₂-VASc Score is more likely used as stroke risk assessment. HAS-BLED score is widely supported by major international guidelines, but mainly for atrial fibrillation [16, 18, 20, 22]. Using these scoring systems can help clinicians assess the bleeding risk in patients receiving anticoagulation therapy to prevent thromboembolism.

This case illustrates the complex interplay between refractory supraventricular tachycardia, structural heart disease, and thromboembolic complications leading to acute limb ischemia (ALI). While incessant SVT is not inherently thrombogenic, the presence of pre-existing cardiomyopathy, multiple cardioversions, and delayed anticoagulation significantly heightened the patient's risk of intracardiac thrombus formation and systemic embolization. The development of a left ventricular thrombus and subsequent ALI underscores the critical need for individualized thromboprophylaxis strategies in high-risk arrhythmia patients, particularly when structural abnormalities are present. Early echocardiographic evaluation, timely initiation of anticoagulation guided by imaging findings and clinical risk, and prompt rhythm stabilization through electrophysiological intervention are essential to improve outcomes.

## Data Availability

No datasets were generated or analysed during the current study.

## Abbreviations

ALI: Acute limb ischemia
WPW: Wolff-parkinson-white
SVT: Supraventricular tachycardia
VT: Ventricular tachycardia
VF: Ventricular fibrillation
BPM: Beats per minute
AVRT: Atrioventricular reentrant tachycardia
BB: Beta blocker
CCB: Calcium channel blocker
ECG: Electrocardiography
LV: Left ventricle
ICU: Intensive care unit
EP: Electrophysiological
DC: Direct current
TEE: Transesophageal echocardiography
CT: Computed tomography
ACC/AHA: American college of cardiology/american heart association
ESC: European society of cardiology
AF: Atrial fibrillation
DOAC: Direct oral anticoagulants
CHA₂DS₂-VASc: Congestive heart failure, Hypertension, Age ≥75 years (doubled), Diabetes mellitus, prior Stroke or TIA or thromboembolism (doubled), Vascular disease, Age 65 to 74 years, Sex category (female)
HAS-BLED: Hypertension, Abnormal Renal/Liver Function, Stroke, Bleeding History or Predisposition, Labile INR, Elderly, Drugs/Alcohol Concomitantly
